# Obesity, COVID-19 and immunotherapy: the complex relationship!

**DOI:** 10.2217/imt-2020-0178

**Published:** 2020-07-17

**Authors:** Carmine Finelli

**Affiliations:** ^1^Department of Internal Medicine, Ospedale Cav R Apicella, ASL Napoli 3 Sud, Via di Massa, 1, 80040 Pollena, Napoli, Italy; ^2^COVID Hospital Boscotrecase, ASL Napoli 3 Sud, Via Lenza, 3, 80042 Boscotrecase, Napoli, Italy

**Keywords:** COVID 19, immune checkpoint inhibitors, immunomodulation, immunopathology, immunotherapy, inflammatory cytokine neutralization, obesity

The coronavirus disease 2019 (COVID-19) is the third known coronavirus after SARS-coronavirus (SARS-CoV) and middle east respiratory syndrome-coronavirus (MERS-CoV) that was first described in late December 2019, until the epidemic began in Wuhan, China, and induces extreme respiratory disease and human pneumonia-like infection [1]. The pandemic spread of novel coronavirus (COVID-19) has been declared a global health emergency by the WHO.

Older adults and people of all ages who may have significant underlying health conditions – associated with obesity – could be at increased risk from COVID-19 for acute disease. Although general obesity is a risk factor for many disorders, several clinical trials have found that the concentration of visceral fat is more strongly related to different health problems, such as coronary disorders, insulin resistance and Type 2 diabetes mellitus [2]. Asthma, chronic lung disorder and cardiovascular disease are the main three health disorders for those infected with COVID-19.

## Obesity & COVID-19

It is still too premature to have concrete data to support this in this pandemic, it is fair to expect that certain patients with obesity – particularly extreme obesity with a BMI over 40 – may have multiple health issues connected to obesity that could be linked to a more severe COVID-19 disease pathway. In the intensive care setting, patients with severe obesity are typically a more challenging population to manage and can struggle to survive if they undergo a significant illness, especially a respiratory infection such as COVID-19. For example, asthma, restrictive lung disease or obstructive sleep apnea could influence the respiratory function of obese patients [3]. Many obese patients experience at least one comorbidity linked to obesity, with Type 2 diabetes and cardiovascular disease being the most severe. Many obesity-related comorbidity include hyperlipidemia, chronic kidney failure, cancer with a cancer history and nonrheumatoid arthritis. This list of chronic diseases details the number of disorders we see every day among our patients that provide obesity treatment [4].

Therefore, obesity poses an elevated risk of severe infection with COVID-19, which may contribute to the need for mechanical ventilation in intensive care units and in the high incidence of mortality with premature death [5]. There are several underlying mechanisms: alteration in respiratory performance, involvement in comorbidities such as diabetes, hypertension, asthma or obstructive sleep apnea, inevitably insufficient and abnormal immunological responses, likely exacerbated by ectopic intrathoracic fat depots. Such results need improved prevention and curative interventions in obese patients in order to reduce the likelihood of relapse to an adverse outcome in COVID-19 cases. Obesity assumes a significant part in the pathogenesis of infection with COVID-19. Indeed, the immune system, which is a main factor in COVID-19 pathogenesis, also plays a key role in inflammation of obesity-induced adipose tissue [6].

We also think it is very significant, and especially with regard to COVID-19, that people who have increased waistlines are likely to have elevated inflammatory markers sometimes. For example, an overactive immune system can result from the so-called inflammatory outbreak, and those individuals with increased waistlines may have elevated levels of inflammatory markers such as CRP, IL-6 or IL-1 [7]. In fact, the understanding of adipose tissue as an inert storage depot started to shift. Nevertheless, evidence began to accumulate that obesity, and especially visceral fat, is correlated with low-grade inflammation due to the elevated production of multiple adipocyte pro-inflammatory cytokines and their related macrophages. Some of those cytokines were often named ‘adipokines’, like leptin, TNF-α, IL-6 [2]. Which is as the theory of obesity blends magnificently with the developing inflammatory theory of COVID-19 pathology, it also links evidence of higher morbidity and mortality in marginalized ethnic and socio-economic groups where food deprivation, obesity and metabolic syndrome are still common [8].

## COVID-19 immunopathology & immunotherapy

Immunotherapy is an important method of treatment to combat viral infections. Most attempts at immunotherapy have been effective in combating related COVID-19 viruses such as SARS-CoV and MERS-CoV, another coronavirus. Many vaccinations and applications for monoclonal antibody are the key approaches in this context. In addition, according to current evidence in the battle toward viral infections such as Ebola, influenza, SARS and MERS, plasma exchange will possibly reduce the viral load and mortality of diseases [9,10].

For all SARS-CoV and SARS-CoV-2 viruses, reaching the host cells is regulated by receptor-binding domain (RBD) association for S protein on the outer-membrane of the virus and angiotensin-converting enzyme 2 on the cell. Such proteins will also be the key possible targets for immunotherapy [1,7]. The awareness of MERS-CoV and SARS-CoV immunotherapies in recent years may expand prospects for successful use of the same therapies for novel coronavirus [3].

As can be seen by the fairly strong identification of RBD in COVID-19 and SARS-CoV, new monoclonal antibodies that could directly attach to COVID-19 RBD need to be established. Some of the most powerful SARS-CoV-specific neutralizing antibodies (e.g., m396, CR3014) targeting the SARS-CoV angiotensin-converting enzyme 2 binding site failed to attach COVID-19 spike protein, suggesting that the variation in SARS-CoV RBD and COVID-19 is distinct [7].

Assuming that both SARS-CoV and COVID-19 have the same virus entry receptor, possible biotherapeutics to inhibit SARS entry may be extrapolated for COVID-19 application. Monoclonal antibodies are favored by immunotherapy strategies to prevent the virus’ attachment or entry because of their precision, purity, low risk of blood-borne pathogen infection and protection compared with serum therapy and preparations for intravenous immunoglobulins [3].

The positive findings in monoclonal antibodies attacking spike protein in SARS-CoV and MERS-CoV motivate researchers to use it in combating COVID-19. Monoclonal antibody mixture or the combination of multiple monoclonal antibodies that identify various epitopes on the viral surface may help to neutralize the virus [3].

Many targets that appear important in immunotherapy of COVID-19 are cytokines. The specificity of IL-6 in COVID-19 among cytokines comes from the fact that increased IL-6 is associated with the intensity of the inflammatory cytokine storms. Therefore, targeting tocilizumab and siltuximab monoclonal antibodies to IL-6 and its receptor (IL-6R) could alleviate cytokine-related symptoms in serious COVID-19 patients [11] and in obese patients with COVID-19 who may have elevated levels of inflammatory markers such as IL-6.

Analysis of T cells in obese mice has led researchers to recognize that most of them are ‘depleted’, a concept of immunology that suggests slow proliferation and that they do not produce chemical messengers for contact with other immune cells [12]. Moreover, in obesity, T cells are thought to release higher levels of programmed cell death-1 (PD-1) protein, which prevents the killing of tumor cells by cytotoxic T cells, thereby facilitating the development and growth of tumors [13]. Immunotherapy researchers are aware of PD-1 and it is PD-L1 ligand and a range of ‘immune checkpoint inhibitors’ have been developed [14]. At this point the connection between obesity and immunotherapy is becoming clearer. The devices with which tumors grow, and probably in viral infection such us COVID-19, in an overweight body are the source of immunomodulatory medications. The presence of PD-1 on the outside of T cells is beyond question, a result of obesity [15].

Acquired cellular immune surveillance is a precisely controlled series of processes and activities affecting particular T lymphocyte populations and subpopulations. The main obligation for the cytotoxic removal of virally compromised and tumor cells rests with CD8^+^ CD3^+^ TcR^+^ [16]. One part of the regulatory arm of cellular immunity results in ‘exhausted T cells’ (Tex), which mainly belong to the CD8^+^ population and occur in persistent high-grade viral infection, such as the COVID-19 [17]. Tex is broadly divided into two subsets: at the initial and intermediate stage, they retain quasi-normal mitochondrial spare respiratory capacity, and are generally responsive to PD-1 blockade of the kind outlined above [18,19]. Tex at the more advanced level, has lower mitochondrial spare respiratory capacity and is terminally depleted and mostly unresponsive to PD-1 checkpoint immunotherapy blockade [20]. Generally speaking, the development of Tim-3 in Tex is similar to that of PD-1, as well as the output of IL-10, TNF-α and other cytokines that mediate apoptotic T cytopenia in some of these patients [21–23].

In patients with reported COVID-19, their data point to major T cytopenia in CD4^+^ and CD8^+^ T cells in circulation [24]. Such patients’ serum had considerably elevated amounts of IL-6, IL-10 and TNF-α. More analyzes found a steady rise in the subpopulation PD-1^+^ CD8^+^ and Tim-3^+^ CD8^+^ as patients progressed from prodromal to symptomatic COVID-19 needing intensive treatment [25,26]. Taken together, the profile mentioned indicates Tex emerging in patients with verified COVID-19.

In view of the fact that immune checkpoint inhibitors immunotherapy may restore cellular immunocompetence, as it previously proposed in the case of influenza infection, the patient undergoing immune checkpoint blockade could be more immunocompetent than cancer patients undergoing chemotherapy [27,28].

This immunotherapy focused on inflammatory cytokine neutralization, immunomodulation and passive viral neutralization may only decrease inflammation, inflammation-associated lung injury, or viral load, but can also avoid acute hospitalization and mechanical ventilation dependency, all of which are restricted options [29].

## Conclusion

At this point, reducing the impact of health inequality on disease occurrence is impossible, not to mention the basic genetic rules for both obesity and morbidity and mortality correlated with COVID-19. Improvements in government policies based on enhancing population nutrition, which not only saves hundreds and even thousands of lives around the world in the coming months, should be expected, But still discourage future pandemics from swamping public systems that are already overrun with people with severe food shortages [30].

What the entire crisis has brought to our notice are the very disturbing health gaps that tend to grow year after year in many countries across the world. We hope that this pandemic will render any health gaps easy for everyone.

While no study has been done, and no vaccinations or antiviral therapeutic agents have been licensed to treat COVID-19 until now, the vaccinations may be effective due to the unique nature of the virus. In fact, current data on SARS-CoV and MERS-CoV immunotherapy carries the potential for usage in COVID-19.

Despite significant progress in the production of a monoclonal antibody-based passive immunotherapy for infection with coronavirus, there is no monoclonal antibody on the market. What limit the use of antibodies is that large-scale production of monoclonal antibodies is laborious, expensive and time-consuming for clinical application. Therefore, it is imperative to plan and build advanced protein development platforms and expression systems to provide effective monoclonal antibodies at an acceptable cost in a short time.

While there is an abundance of data in obesity-related inflammation and related diseases affecting a disordered immune system, further research is urgently needed to identify novel approaches for the prevention and treatment of obesity-linked diseases like COVID-19. Research demonstrates a combination of both immunotherapy and lifestyle-dependent treatments can reduce prevalence and improve obesity-related malignancy outcomes. New findings are also updated to exploit obesity for favorable benefits, although associated with several harmful cancer causes, provided the involved mechanisms are handled properly. In fact, a large body of literature relates to a ‘meta-inflammatory’ state of obesity associated with a deficit in the immune response. It is important to understand how obesity shapes immune responses and how obesity can change both the responses to immunotherapy and the potential toxicity. Therefore, all this suggests a surprisingly positive association between obesity, COVID-19 and immunotherapy.

More studies may help to establish how obesity enhances PD-1^+^ CD8 T cells in a significant number of COVID-19 patients, and whether this mechanism often leads to a decreased occurrence of COVID-19 in obese patients.
